# Low CCR7-Mediated Migration of Human Monocyte Derived Dendritic Cells in Response to Human Respiratory Syncytial Virus and Human Metapneumovirus

**DOI:** 10.1371/journal.ppat.1002105

**Published:** 2011-06-23

**Authors:** Cyril Le Nouën, Philippa Hillyer, Christine C. Winter, Thomas McCarty, Ronald L. Rabin, Peter L. Collins, Ursula J. Buchholz

**Affiliations:** 1 Laboratory of Infectious Diseases, National Institute of Allergy and Infectious Diseases, National Institutes of Health, Bethesda, Maryland, United States of America; 2 Center for Biologics Evaluation and Research, U.S. Food and Drug Administration, Bethesda, Maryland, United States of America; University of Pennsylvania, United States of America

## Abstract

Human respiratory syncytial virus (HRSV) and, to a lesser extent, human metapneumovirus (HMPV) and human parainfluenza virus type 3 (HPIV3), can re-infect symptomatically throughout life without significant antigenic change, suggestive of incomplete or short-lived immunity. In contrast, re-infection by influenza A virus (IAV) largely depends on antigenic change, suggestive of more complete immunity. Antigen presentation by dendritic cells (DC) is critical in initiating the adaptive immune response. Antigen uptake by DC induces maturational changes that include decreased expression of the chemokine receptors CCR1, CCR2, and CCR5 that maintain DC residence in peripheral tissues, and increased expression of CCR7 that mediates the migration of antigen-bearing DC to lymphatic tissue. We stimulated human monocyte-derived DC (MDDC) with virus and found that, in contrast to HPIV3 and IAV, HMPV and HRSV did not efficiently decrease CCR1, 2, and 5 expression, and did not efficiently increase CCR7 expression. Consistent with the differences in CCR7 mRNA and protein expression, MDDC stimulated with HRSV or HMPV migrated less efficiently to the CCR7 ligand CCL19 than did IAV-stimulated MDDC. Using GFP-expressing recombinant virus, we showed that the subpopulation of MDDC that was robustly infected with HRSV was particularly inefficient in chemokine receptor modulation. HMPV- or HRSV-stimulated MDDC responded to secondary stimulation with bacterial lipopolysaccharide or with a cocktail of proinflammatory cytokines by increasing CCR7 and decreasing CCR1, 2 and 5 expression, and by more efficient migration to CCL19, suggesting that HMPV and HRSV suboptimally stimulate rather than irreversibly inhibit MDDC migration. This also suggests that the low concentration of proinflammatory cytokines released from HRSV- and HMPV-stimulated MDDC is partly responsible for the low CCR7-mediated migration. We propose that inefficient migration of HRSV- and HMPV-stimulated DC to lymphatic tissue contributes to reduced adaptive responses to these viruses.

## Author Summary

The respiratory viruses human respiratory syncytial virus (HRSV) and, to a lesser extent, human metapneumovirus virus (HMPV) and human parainfluenza virus (HPIV3), can re-infect humans throughout life without significant antigenic change, suggesting that immunity to these viruses is incomplete. In contrast, re-infection by influenza A virus (IAV) depends on antigenic change, suggestive of more complete immunity. Dendritic cells (DC) take up virus antigen at the site of infection, undergo maturation, and migrate to the lymphatic tissue to present antigen to T lymphocytes, orchestrating the immune response. In response to antigen uptake, DC switch chemokine receptors on their surface, decreasing expression of receptors for inflammatory chemokines in the infected tissue, and increasing expression of CCR7, the sole chemokine receptor that directs DC to migrate to lymphatic tissue. By stimulating human DC *in vitro*, we found that, in contrast to HPIV3 and IAV, HMPV and HRSV did not efficiently induce the switching of these surface receptors. In cell migration assays, we showed that, compared with IAV-treated DC, HRSV- or HMPV-treated DC migrated less efficiently to CCL19, a chemokine that directs T cell migration to lymphatic tissue. Thus, during infection with HRSV and HMPV, inefficient migration of DC to the lymphatics could reduce the adaptive immune response to these viruses.

## Introduction

The paramyxoviruses human respiratory syncytial virus (HRSV), human metapneumovirus (HMPV) and human parainfluenza virus type 3 (HPIV3) are common respiratory pathogens. HRSV is the most important viral agent of severe pediatric respiratory tract disease worldwide [1], [2], followed by HPIV3 [3], [4] and HMPV [5], [6], [7], [8]. The orthomyxovirus influenza virus type A (IAV) infects and causes respiratory tract disease in all age groups [9], [10], [11].

These human respiratory viruses share a tropism for the respiratory epithelium and have overlapping spectra of disease, ranging from rhinitis to bronchiolitis and pneumonia [12], [13]. IAV usually induces long-term immunity following infection, such that re-infection depends on significant antigenic change [14], [15]. In contrast, HMPV, HRSV and HPIV3 are able to symptomatically re-infect humans throughout life without significant antigenic change. This is particularly common with HRSV. Glezen and colleagues followed children from birth, and found that more than two-thirds were infected with HRSV during the first year of life, and almost half of these individuals were re-infected during each of the next two years [16]. In experimental infections of adults, typically 50–80% of subjects are re-infected with HRSV, and the majority has acute illness [17]. In another study, adults were challenged at intervals of 2–6 months over a period of 26 months with the same HRSV isolate, with the result that 73% were infected at least twice and 43% at least three times, and more than half of these infections were symptomatic [18]. These observations have been widely interpreted to indicate that HRSV in particular blunts or skews the immune response, resulting in suboptimal protection.

Antigen-presenting dendritic cells (DC) are critical for a functional adaptive immune response. During a lower respiratory tract infection, the number of DC in the bronchi and lung increases by chemotactic influx of precursors that originate primarily from circulating monocytes [19], [20], [21], [22]. Migration to non-lymphoid peripheral tissues such as the lung is mediated by so called “inflammatory” chemokine receptor-ligand pairs, including CCR1-CCL3/MIP-1α, CCR2-CCL2/MCP-1 or CCR5-CCL5/RANTES. Exposure of DC to antigen in peripheral tissue initiates DC maturation. During maturation, DC increase the surface expression of co-stimulatory molecules such as CD38, CD40, CD80 CD86, and CD83 [23], [24]. DC also change their expression of cell surface chemokine receptors: expression of CCR1, CCR2, and CCR5 is reduced, reducing responsiveness to inflammatory chemokines, and expression of CCR7 is increased [25], [26]. CCR7 has two specific ligands, CCL19 and CCL21, which are expressed by endothelial cells in lymphatic venules, in high endothelial venules (HEV) in lymph nodes, and in the T cell zone of lymphoid organs [27], [28], [29]. CCL19 and CCL21 direct migration of maturing, CCR7-expressing DC through the afferent lymphatics to the draining lymph nodes, and control DC positioning within defined functional lymphoid compartments [25], [26], [30], [31] for efficient interaction with naïve and/or antigen-specific memory T lymphocytes. DC have a key role in determining the magnitude and quality of the adaptive immune response.

We previously reported that HMPV, HRSV, and HPIV3 induce low-to moderate levels of human monocyte-derived dendritic cell (MDDC) maturation, cytokine/chemokine expression, and CD4 T cell proliferation, with the magnitude increasing slightly in the order HRSV, HMPV, and HPIV3 [32], [33]. MDDC generated *in vitro* from primary human monocytes by treatment with IL-4 and GM-CSF represent an appropriate model for lung DC because monocytes give rise to myeloid DC in the resting lung [34] and mucosa [35], and are phenotypically and functionally similar to DC located at sites of inflammation *in vivo* [36]. In the present study, we expanded our previous findings by screening MDDC for expression of genes related to maturation in response to HMPV, HPIV3, HRSV and, for comparison, IAV. We found that CCR7 mRNA and protein expression were substantially increased in response to HPIV3 and IAV, but minimally increased in response to HMPV and HRSV. These differences detected by qRT-PCR and flow cytometry were functionally relevant, since MDDC stimulated with HMPV or HRSV were less efficient in their migration along a CCR7 concentration gradient than IAV- and HPIV3 stimulated MDDC. Secondary stimulation of HRSV- or HMPV-exposed MDDC with the strong DC activator LPS enhanced CCR7 expression and *in vitro* migration, suggesting that suboptimal stimulation, rather than inhibition, is responsible for this poor-migration phenotype. Finally, we provide evidence that low CCR7 expression by MDDC in response to HRSV and HMPV is at least partly due to the low level of expression of pro-inflammatory cytokines (TNF-α, IL-1α and IL-6).

## Materials and Methods

### Ethics statement

Elutriated monocytes were obtained from healthy donors at the Department of Transfusion Medicine of the National Institutes of Health, under a protocol (99-CC-0168) approved by the IRB of the Clinical Center, NIH. Written informed consent was obtained from all donors.

### Virus stock preparation

Recombinant (r) HMPV (strain CAN97-83), rHRSV (strain A2) and rHPIV3 (strain JS) with or without the GFP gene were described previously [12], [37], [38]. The present study employed a genetically “stabilized” version of rHMPV, in which the SH gene was modified to silently remove tracts of A and T residues that had been sites of spontaneous mutations during passage *in vitro* [39]. Human Influenza/A/Udorn/72, a wildtype virus of subtype H3N2, was used as control.

All viruses were grown on Vero cells and purified by centrifugation through sucrose step gradients as described previously [32]. Sucrose purified viruses were pelleted by centrifugation to remove sucrose. Virus pellets were resuspended in Advanced RMPI 1640 (Invitrogen, Carlsbad, CA) supplemented with 2 mM L-glutamine (aRPMI), and aliquots were snap frozen and stored at −80°C until use. Virus titers were determined by immuno-plaque assay on Vero cells under methylcellulose overlay (containing trypsin for titration of rHMPV and IAV) as described previously [37]. In some experiments, UV-inactivated viruses were included as controls which were prepared using a Stratalinker UV cross-linker (Agilent, Santa Clara, CA) at 0.5 J/cm^2^. Complete inactivation was monitored by plaque assay (limit of detection: 5 plaque forming units per mL).

### Generation of immature monocyte-derived DC

Elutriated monocytes were obtained from healthy donors at the Department of Transfusion Medicine of the National Institutes of Health, under a protocol (99-CC-0168) approved by the IRB of the Clinical Center, NIH. As previously described [32], monocytes were subjected to CD14+ sorting on an Automacs separator (Miltenyi Biotec, Auburn CA), and cultured in presence of recombinant human IL-4 (R&D Systems, Minneapolis, MN) and recombinant human granulocyte-macrophage colony-stimulating factor (GM-CSF, Bayer Healthcare, Wayne, NJ) for 7 days to generate immature MDDC. These were confirmed by flow cytometry to be CD14^−^, CD38^−^, CD80^low^, CD86^low^, CD40^low^, CD54^low^.

### MDDC treatments

Immature MDDC were seeded in 12-well plates at 6×10^5^ cells per well and were infected with live virus at an MOI of 3 PFU/cell, or with an equivalent amount of UV-inactivated virus, stimulated with 1 µg/ml of the superantigen *Staphylococcus* enterotoxin B (SEB; Sigma, St Louis, MO) or with 1 µg/ml of lipopolysaccharide (LPS) from Escherichia Coli O55:B5 (Sigma). The infectivity of rgHMPV, rgHRSV and rgHPIV3 for MDDC was similar (approximately 3–5% of GFP+ MDDC at 24 or 48 h post infection, no significant differences at the p≤0.05 confidence level for any of the data sets of this study). In some experiments, immature MDDC infected with rgHMPV, rgHRSV, rgHPIV3 at an input MOI of 3 PFU/cell were further stimulated 4 to 6 h later with 1 µg/ml of LPS or 150 IU of Interferon (IFN)-β (PBL Interferon source, Piscataway, NJ) or with a cocktail of pro-inflammatory cytokines of 6 ng/ml TNF-α, 10 ng/ml IL-6 and 0.36 ng/ml IL-1α (R&D systems). All inoculations or stimulations were performed in advanced RMPI 1640 (Invitrogen) supplemented with 10% heat-inactivated FBS (Hyclone, Logan, UT), 2 mM L-glutamine (Invitrogen), 200 U/ml penicillin, and 200 µg/ml streptomycin (Invitrogen) at 37°C in 5% CO_2_.

### Reverse transcription and quantitative PCR

Cell-associated RNA was isolated using the RNeasy mini kit (Qiagen) as recommended by the manufacturer and treated with DNAse I to remove residual genomic DNA. Analysis was done in two ways. The first involved a custom made low-density Taqman gene array containing 62 genes. Here, 1 µg of isolated RNA was reverse transcribed using SuperScript II (Invitrogen) in a 50 µl mix using random primers, and each cDNA mix was loaded onto an array in triplicate. The second method involved individual RT-PCR reactions. Here, 600 ng of isolated RNA was reverse transcribed using superscript II (Invitrogen) in a 25 µl mix using random primers. The reverse transcription product was diluted three-fold, and two µl of the diluted cDNA mix were used in each quantitative TaqMan PCR (Applied Biosystems, CA) for quantification of the targets of interest, namely CCR1 (Hs00174298_m1), CCR5 (Hs00152917_m1) and CCR7 (Hs00171054_m1). qPCR results were analyzed using the comparative threshold cycle (ΔΔC_T_) method, normalized to 18S rRNA and expressed as fold change over mock.

### Flow cytometry analysis of CCR1, 2, 5 and 7 expression

To determine the surface expression level of chemokine receptors, cells were stained with allophycocyanin (APC)-conjugated anti-human mAbs [anti-CCR1 (CD191, clone 53504), anti-CCR2 (CD192, clone 48607), anti-CCR5 (CD195, clone 2D7), anti-CCR7 (CD197, clone 2H4) (BD Biosciences, San Jose, CA)]. Isotype-matched mAbs were included as controls. Propidium iodide staining was used to exclude dead cells from further analysis. At 48 h post infection, the median viability of MDDC from six independent experiments was 85% for HMPV-, 86% for HRSV-, and 82% for HPIV3-exposed MDDC, reflecting the anti-apoptotic effects of virus-induced DC maturation [32]. In order to avoid interference, CCR1, 2, 5 and 7 expression was analyzed individually. At least 20,000 events were acquired using a FACSCalibur flow cytometer (BD Biosciences) and analyzed using FlowJo version 8.8.6 software (Tree Star, Inc., Ashland, OR).

### Chemotaxis assay

After 48 h stimulation, migration of the virus-stimulated MDDC in response to a CCL19 concentration gradient was evaluated using polycarbonate 5-µm diameter pore size transwells (Corning, Lowell, MA). 1×10^5^ live MDDC were seeded in the upper chamber, and incubated in presence or absence of CCL19 (1 µg/ml, (R&D Systems, Minneapolis, MN) in the lower chamber. Duplicate wells were used for each condition. After 3 h incubation, MDDC from the lower chamber were harvested, and the cell density of live cells was determined using a FACS Calibur flow cytometer (BD Biosciences). For each sample, data acquisition was performed for 1 min at constant flow using 200 µl final volume. Forward scatter, side scatter, live/dead staining, and GFP expression were analyzed using FlowJo version 8.8.6 software (Tree Star, Inc., Ashland, OR). The average number of MDDC specifically migrating in response to CCL19 was calculated as follows: (Average number of stimulated MDDC migrated in the presence of CCL19) – (Average number of stimulated MDDC migrated in the absence of CCL19).

### Statistical analysis

Data sets were assessed for significance using parametric one-way repeated measures ANOVA with the Tukey post hoc tests for normally distributed data sets or the non-parametric Friedman test with Dunns post hoc test. A log_10_ transformation was applied to data sets when necessary to obtain equal standard deviation among groups, a necessary requirement of both tests. Statistics were performed using Prism 5 (GraphPad Software, Inc, San Diego, CA). Data were only considered significant at P<0.05. Analysis of CCR5 and CCR7 expression: To account for the smaller data set of the IAV control (n = 8 donors, except for IAV, n = 6 donors), data were analyzed using an unbalanced repeated measures ANOVA (JMP version 8.0.2; SAS, Cary, NC).

## Results

### Gene expression survey of human MDDC stimulated with rHMPV, rHRSV, rHPIV3 and IAV

We used RT-qPCR to survey maturation-related gene expression in MDDCs from 3 donors 24 h after exposure to either the superantigen SEB or to purified live or UV-inactivated rHRSV, rHMPV, rHPIV3, or IAV (Fig. S1A and B). In general, all four live viruses induced the up-regulation of the same array of genes but differed in the intensity of up-regulation increasing in the order rHRSV<rHMPV<IAV<rHPIV3. The donors also had substantial responses to UV-inactivated IAV, but weak responses to UV-inactivated rHMPV, rHRSV or rHPIV3. Donors 1 and 2 were refractory to stimulation by rHMPV and rHRSV, respectively.

**Figure 1:**
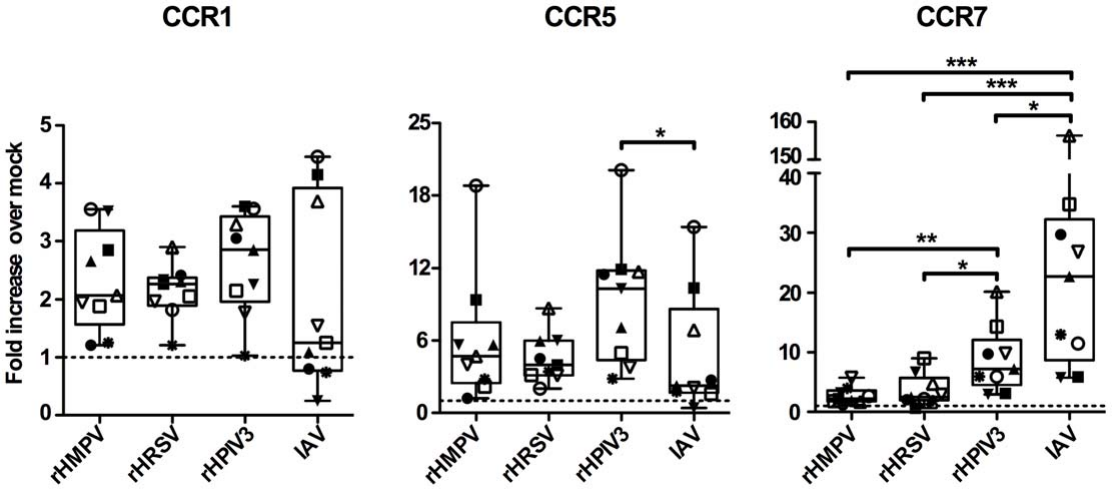
CCR1, 5 and 7 gene expression of MDDC stimulated with rHMPV, rHRSV, rHPIV3, or IAV. Immature MDDC (n = 9 donors) were infected with rHMPV, rHRSV, rHPIV3, or influenza/A/Udorn (IAV). Twenty-four h post infection, total cellular RNA was prepared and reverse-transcribed using random primers, and the cDNA analyzed in triplicate by qPCR using TaqMan PCR assays. qPCR results were analyzed using the comparative threshold cycle (ΔΔC_T_) method, normalized to 18S rRNA. The results are expressed as fold-increase over mock. Statistical differences are indicated by asterisks (* P≤0.05, ** P≤0.01, *** P≤0.001, Materials and Methods).

Among the genes surveyed, expression of CCR7 mRNA was substantially increased in response to IAV and rHPIV3, but not in response to rHMPV and rHRSV (Fig. S1A). Based on these preliminary results, we analyzed CCR7 mRNA expression by qPCR in additional donors (total n = 9, Fig. 1), and found that, while IAV and HPIV3 induced a strong increase of CCR7 mRNA (median increases of 23-fold and 7.2 fold, respectively), HRSV and HMPV only induced a 2.2- and 2.5-fold increase compared to mock treated cells. The effects of HMPV and HRSV on CCR7 expression were significantly smaller compared to HPIV3 and IAV (Fig. 1). By contrast, expression of CCR1 and CCR5 mRNA was increased in response to all viruses, but without any statistical difference between the viruses, except that the CCR5 mRNA expression was significantly different between rHPIV3 and IAV (Fig. 1). Because CCR7 has a unique role in DC migration towards lymph nodes and the subsequent adaptive response [26], we explored the effect of these viruses on MDDC chemokine receptor expression and migration.

### MDDC treated with rgHMPV or rgHRSV do not efficiently change cell surface chemokine receptor expression

**Figure 2:**
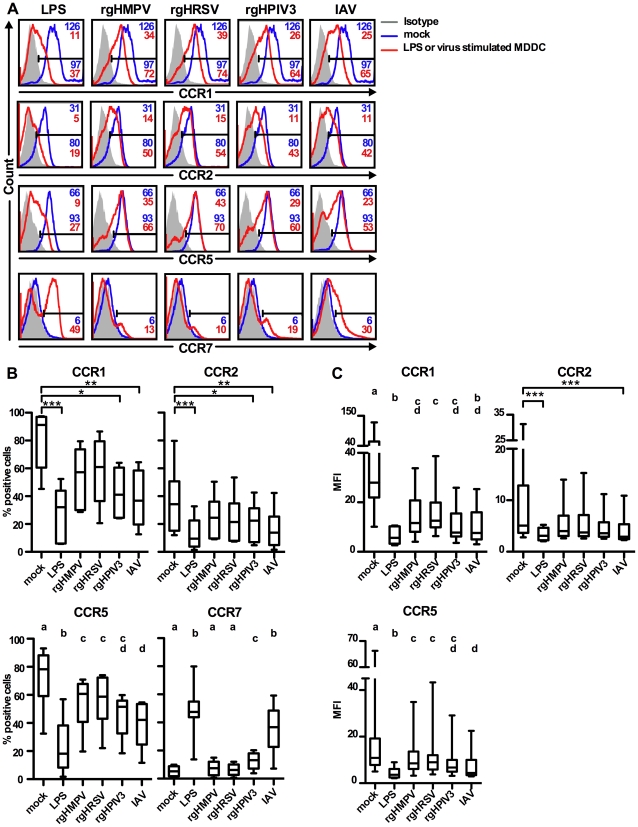
Cell surface expression of the chemokine receptors CCR1, 2, 5 and 7. Immature MDDC were stimulated with LPS or infected with rgHMPV, rgHRSV, rgHPIV3, or influenza/A/Udorn (IAV) at an MOI of 3 PFU/cell. 48 h post-infection, the surface expression levels of CCR1, 2, 5 and 7 were assessed by flow cytometry. (A) Surface expression of CCR1, 2, 5, and 7 from a representative donor. The MFI (top right corner) and % positive (bottom right) of each cell population are indicated for each histogram, with red indicating the treated population and blue indicating the mock-treated control. Note that values of MFI are not given in the case of CCR7 because only a small percentage of treated MDDC expressed this molecule at the cell surface. (B, C) Summary of data expressed as % positive cells (B) and as MFI (C). MFI is not shown for CCR7, as explained for (A). CCR1, 2: n = 6 donors; CCR5, 7: n = 8 donors, except n = 6 donors for IAV. The box plots show the median (horizontal line), flanked by the 2^nd^ and 3^rd^ quartile. The outer bars show the range of values. Statistical differences are indicated by asterisks (* P≤0.05, ** P≤0.01, *** P≤0.001, Materials and Methods). CCR5 and 7 expression: To account for the smaller data set of the IAV control (n = 8 donors, except for IAV, n = 6 donors), data were analyzed using an unbalanced repeated measures ANOVA (JMP 8.0.2; SAS, Cary, NC). Treatments sharing the same lower case letters do not differ significantly at the p≤0.05 confidence level, see Materials and Methods.

We next used flow cytometry to measure surface expression of CCR1, 2, 5, and 7 on MDDC 48 h after exposure to the different viruses (Fig. 2). We included CCR2 in this analysis since, like CCR1 and CCR5, it directs monocytes and DC to inflamed tissue and is down-regulated during DC maturation. LPS was used as positive control because it strongly activates DC [40], [41]. Fig. 2A shows primary data for a representative donor, and Fig. 2B–C show the compiled results for six to eight donors. In this and all subsequent experiments, we used versions of rHMPV, rHRSV, and rHPIV3 that express GFP from an added gene (rgHMPV, rgHRSV, and rgHPIV3, respectively).

In mock-treated MDDC, substantial subpopulations of cells expressed CCR1 (median 91% of total), CCR2 (34%), and CCR5 (75%), and were CCR7-negative or low (Fig. 2A, B, C). High CCR1/2/5 and low CCR7 values would be typical for immature DC residing in peripheral tissue. As expected, LPS treatment induced a significant down-regulation of CCR1 (32%), CCR2 (9%), and CCR5 (24%), and up-regulation of CCR7 (48% positive cells) (Fig. 2A, B, C). Stimulation of MDDC with rgHPIV3 or IAV also induced a significant decrease in frequency of cells expressing the inflammatory chemokine receptors CCR1, 2, and 5 compared to mock-treated cells (Fig. 2B). However, only IAV significantly decreased all median fluorescence intensities (MFIs) (Fig. 2C).

In contrast, stimulation with rgHMPV or rgHRSV had only moderate effects on chemokine receptor surface expression. Cell surface expression of CCR1 and 2 decreased after stimulation with rgHRSV and rgHMPV, but the difference compared to mock-treated MDDC was not significant (Fig. 2B and C), except for the MFI of CCR1 (Fig. 2C). Stimulation with rgHMPV or rgHRSV reduced the percentage of CCR5+ MDDC significantly compared to mock-treated MDDC, but treatment with IAV reduced CCR5 expression significantly more than rgHMPV or rgHRSV treatment (Fig. 2B). CCR5 expression of rgHPIV3 stimulated MDDC was intermediate between HMPV and HRSV on the one hand, and IAV on the other hand, with no significant differences to any of the viruses (Fig. 2C).

The limited down-regulation of CCR1, 2, and 5 in response to rgHMPV and rgHRSV was coupled with a weak increase of CCR7 expression occurring on only a small subpopulation of cells (Fig. 2B, median 7% CCR7^+^ cells for for rgHMPV, and 6% for rgHRSV, with no statistical difference to mock). Stimulation with rgHPIV3 or IAV was associated with a significantly stronger up-regulation of CCR7 than mock, rgHMPV or rgHRSV stimulation, resulting in 13% and 37% CCR7+ cells, respectively.

Taken together, these results showed that compared to LPS, IAV, and rgHPIV3, stimulation with rgHMPV and rgHRSV induced a smaller down-regulation of surface expression of CCR1, CCR2, and CCR5, and a smaller up-regulation of CCR7 surface expression occurring on a smaller fraction of cells.

#### MDDC treated with HMPV or HRSV migrate poorly to the CCR7 ligand CCL19

**Figure 3:**
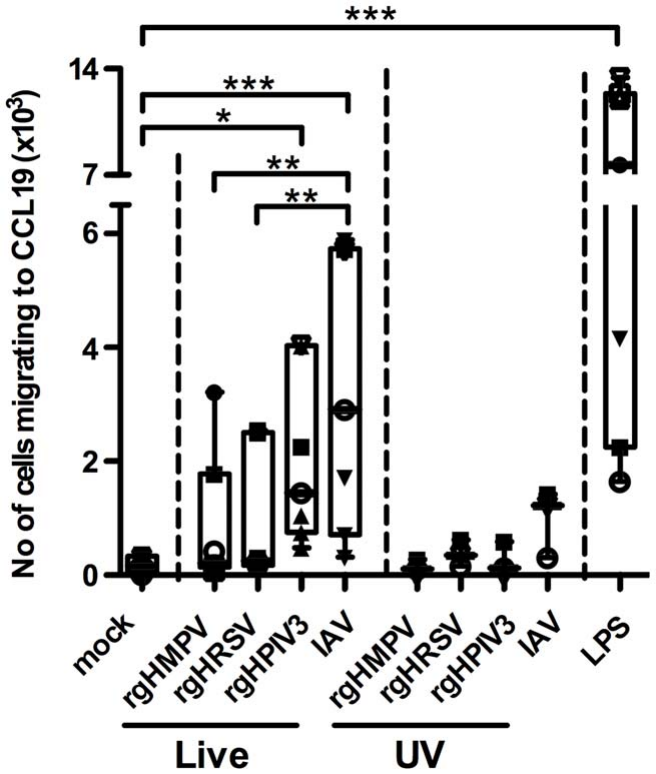
MDDC stimulated with IAV migrate more efficiently to a CCL19 concentration gradient than HMPV- or HRSV-stimulated MDDC. Immature MDDC were stimulated with LPS or infected with rgHMPV, rgHRSV, rgHPIV3, IAV, or with their UV-counterparts and, 48 h following stimulation, were assessed for the ability to migrate *in vitro* to a CCL19 concentration gradient. This was done using transwell cultures containing a polycarbonate filter with a pore diameter of 5 µm. 1×10^5^ live MDDC were seeded in the upper chamber and incubated in presence or absence of CCL19 in the lower chamber. After 3 h, MDDC from the lower chamber were harvested, and the cell density was determined by flow cytometry. Data were expressed as the average number of MDDC migrating specifically to a CCL19 concentration gradient, calculated as follows: (Average number of stimulated MDDC migrating in the presence of CCL19) – (Average number of stimulated MDDC migrating in the absence of CCL19). The median number of MDDC migrating in absence of CCL19 for the seven analyzed donors was 207, and thus the background was low. The box plots show the median (horizontal line), flanked by the 2^nd^ and 3^rd^ quartile. The outer bars show the range of values. Each donor is represented by an individual symbol. n = 7 donors except for the UV-inactivated viruses, where n = 3 donors. Statistical differences are indicated by asterisks (* P≤0.05, ** P≤0.01, *** P≤0.001, Materials and Methods).

We asked whether the lower level of CCR7 surface expression by MDDC stimulated with rgHRSV or rgHMPV affected their migration in response to the CCR7 ligand CCL19. MDDC were stimulated with LPS or the various live or UV-inactivated viruses and assayed for the ability to migrate along a CCL19 concentration gradient in a trans-well system (Fig. 3). As expected, LPS-stimulated MDDC migrated well, while mock and UV-inactivated virus-stimulated MDDC migrated poorly towards CCL19. There was a small (but not statistically significant) increase in migration for UV-IAV stimulated MDDC (between 302 and 1417 specifically migrating MDDC), commensurate with the slight increase in CCR7 mRNA expression in two out of 3 donors after exposure to UV-inactivated IAV virus (Fig. S1A). Complete UV inactivation of IAV and the other viruses had been verified by titration. Thus, the small stimulatory effect of UV-IAV could not be attributed to partial inactivation of IAV.

Migration of MDDC towards CCL19 was increased for each of the live viruses. However, MDDC stimulated with live rgHMPV and rgHRSV migrated less efficiently towards CCL19 than cells stimulated with rgHPIV3 and IAV (medians: 202, 246, 1445, and 2903 specifically migrating MDDC, respectively). While rgHPIV3- and IAV-stimulated MDDC migrated to CCL19 in significantly higher numbers than mock-stimulated MDDC, migration of rgHMPV- and rgHRSV-stimulated MDDC was not significantly different from that of mock-stimulated MDDC. Migration of IAV-stimulated MDDC was significantly greater than that of rgHRSV- and rgHMPV-stimulated MDDC (p<0.01; ANOVA/Tukey post hoc analysis comparing mock-stimulated MDDC with those stimulated with live virus). In summary, this assay showed that the weaker up-regulation of CCR7 mRNA and surface protein expression in response to rgHMPV and rgHRSV compared to IAV and LPS was associated with less efficient migration in response to CCL19.

### Comparison of chemokine receptor expression on the GFP-positive versus GFP-negative subpopulations of virus-stimulated MDDC

**Figure 4:**
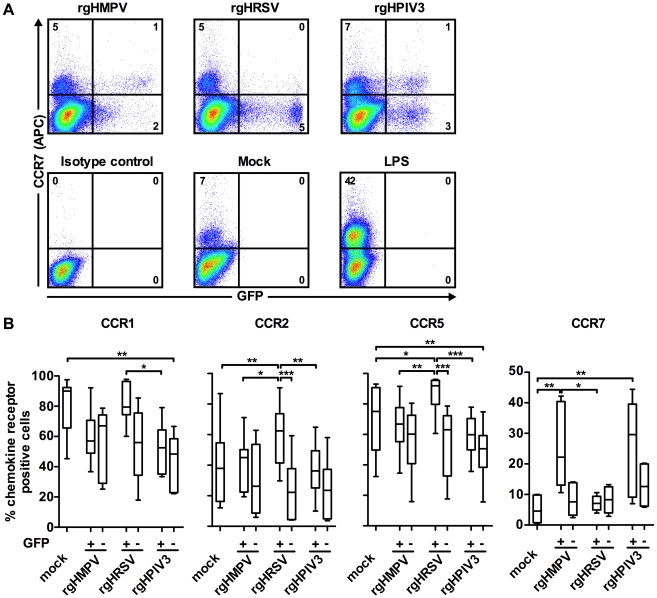
Chemokine receptor surface expression on GFP-positive versus GFP-negative MDDC following inoculation with rgHMPV, rgHRSV and rgHPIV3. 48 h after stimulation with LPS or infection with the indicated viruses, GFP+ and GFP− cells were analyzed by flow cytometry for cell surface expression of the indicated chemokine receptors. (A) Scatter plots of GFP expression and CCR7 surface staining from a typical donor and (B) summary of the data from n = 6 donors. (+) GFP+ population, (−) GFP− population. The box plots show the median (horizontal line), flanked by the 2^nd^ and 3^rd^ quartile. The outers bars show the range of values. Statistical differences are indicated by asterisks (* P≤0.05, ** P≤0.01, *** P≤0.001, Materials and Methods). Note that the statistical differences between the GFP+ cells from a given virus and the GFP− cells from a different virus are not shown.

We used flow cytometry to compare chemokine receptor surface expression on virus-exposed cells that were GFP-positive versus GFP-negative (Fig. 4). We previously showed that, following infection with rgHMPV, rgHRSV, or rgHPIV3 at an MOI of 3, approximately 3–5 % of MDDC were GFP+ at 24 or 48 h post-infection [32]. This was indicative of robust viral gene expression, which was confirmed by RT-qPCR. In the GFP– population, we detected a low level of viral gene expression, suggestive of abortive virus replication [32]. Thus, comparing host gene expression in GFP+ and GFP– cells provides an indication of the effects of a robust versus abortive infection. Fig. 4A shows primary data for GFP expression and CCR7 surface expression for a single donor, and Fig. 4B summarizes data for the expression of CCR1, 2, 5, and 7 for six donors.

After treatment of MDDC with rgHMPV or rgHPIV3, the extent of down-regulation of CCR1, 2, and 5 was similar between the GFP+ and GFP− MDDC (Fig. 4). In contrast, after rgHRSV treatment, these receptors were decreased only in the GFP− cells; indeed, in the GFP+ cells, CCR2 and CCR5 expression was slightly increased compared to mock treated cells. Thus, robust rgHRSV gene expression did not induce the down-regulation of the inflammatory chemokine receptors CCR1, 2, and 5 that normally occurs as part of DC maturation.

CCR7 was expressed at higher levels in the GFP+ cells than in the GFP− cells after treatment with rgHMPV or rgHPIV3, indicating that robust infection by these viruses stimulated rather than inhibited expression (Fig. 4A and B). In contrast, CCR7 expression was not increased in either the GFP+ or the GFP− subpopulations of cells treated with rgHRSV.

### The weak CCR7-driven migration of MDDC treated with rgHMPV and rgHRSV can be increased by secondary stimulation with pro-inflammatory cytokines or LPS

One possible explanation for the weak chemokine receptor modulation and migration by rgHMPV- and rgHRSV-treated MDDC was direct virus-mediated inhibition. Alternatively, it was possible that these viruses were insufficiently stimulatory, perhaps due to the low production of cytokines by virus-treated MDDC as described in our previous study [32]. We therefore investigated whether exposure of virus-stimulated MDDC to secondary stimulation with LPS or to higher concentrations of cytokines would result in more efficient chemokine receptor modulation and migration. We tested possible cytokine and IFN candidates based on the gene expression analysis described above (Fig. S1) and previously published data by ourselves and others [32], [42], [43], [44], [45], [46]. The individual additions of IFN-β, IL-28, IL-29, TNF-α, IL-1α, IL-6 and prostaglandin E2 to virus-treated MDDC had little or no effect on CCR7 mRNA levels or on the ability of MDDC to migrate to a CCL19 concentration gradient (data not shown). These preliminary results confirmed previously published data showing that CCR7 is not an IFN-regulated gene in human or mouse DC [47], [48], [49]. Thus, the poor up-regulation of CCR7 by rgHMPV and rgHRSV is unlikely to be the result of a more stringent IFN antagonism by these viruses.

**Figure 5:**
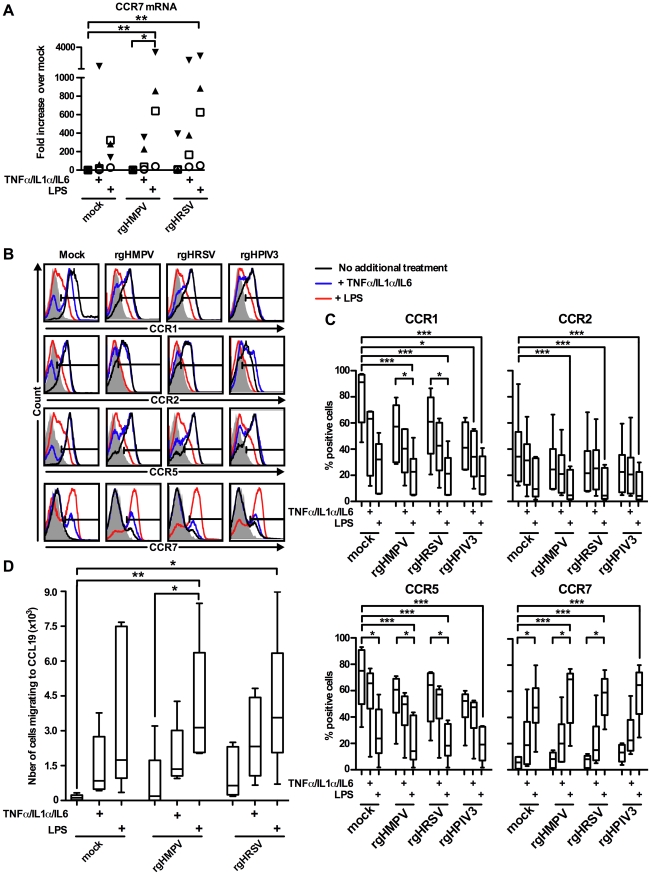
A cocktail of pro-inflammatory cytokines TNF-α/IL-1α/IL-6 partly restores the CCR7-driven migration of rgHMPV- or rgHRSV-stimulated MDDC. MDDC were infected with rgHMPV or rgHRSV and, 4–6 h post-infection, replica MDDC cultures were stimulated with a cocktail of the pro-inflammatory cytokines TNF-α/IL-1α/IL-6 or with LPS. (A) CCR7 mRNA levels in cells harvested at 24 h post-infection were quantified by RT-qPCR using the ΔΔC_T_ method and expressed as fold change over mock. Each symbol represents an individual donor; n = 4 donors. (B, C) Cell surface expression of the chemokine receptors CCR1, CCR2, CCR5 and CCR7 in cells harvested 48 h post-infection were quantified by flow cytometry. Primary data from a representative donor (B) and summary of data from n = 6 donors are shown (C). The box plots show the median (horizontal line), flanked by the 2^nd^ and 3^rd^ quartile. The outer bars show the range of values. (D) MDDC migration to a CCL19 gradient, measured using the assay in Fig. 3, for cells harvested 48 h post-infection. The box plots show the median (horizontal line), flanked by the 2^nd^ and 3^rd^ quartile. The outer bars show the range of values. n = 5 donors. Statistically significant differences induced by cytokine or LPS treatment, and differences of any sample compared to untreated mock-infected cells, are indicated by asterisks (* P≤0.05, *** P≤0.001, see materials and methods for statistical analysis).

We next tested the effect of a cocktail of pro-inflammatory cytokines containing TNF-α, IL-1α and IL-6 on chemokine receptor expression, with each cytokine in concentrations similar to those induced by LPS under our experimental conditions [32]. MDDC (n = 4 donors) were treated with rgHMPV or rgHRSV, and, 4–6 h later, received a secondary stimulation with the cocktail of pro-inflammatory cytokines or with LPS. The expression levels of CCR7 mRNA were quantified 24 h post-infection (Fig. 5A). The secondary treatment with LPS induced a significant (p<0.05) increase of CCR7 mRNA expression in rgHMPV- and rgHRSV-stimulated MDDC. Thus, the relatively low level of expression of CCR7 in MDDC exposed to rgHMPV or rgHRSV was not due to an irreversible block. Following treatment with the cocktail of pro-inflammatory cytokines, there was an increase of CCR7 mRNA in mock-, rgHMPV- or rgHRSV-stimulated MDDC, although there was substantial individual variation and this increase did not reach statistical significance. This suggests that the low level of expression of CCR7 mRNA in MDDC stimulated with rgHMPV or rgHRSV might be partly a consequence of the low levels of TNF-α, IL-1α and IL-6 produced after exposure to rgHMPV or rgHRSV.

To measure cell surface protein expression, MDDC that were treated with rgHMPV or rgHRSV and given a secondary stimulation with the pro-inflammatory cytokine cocktail or LPS, as described above, were analyzed by flow cytometry at 48 h post-infection. Consistent with the results at the mRNA level, stimulation with the proinflammatory cytokine cocktail induced a partial decrease in CCR1, 2 and 5 as well as a partial increase in CCR7 surface expression (Fig. 5B and C; B: 1 representative donor, and C: n = 6 donors). Secondary stimulation with LPS had stronger effects in all cases.

We also evaluated replicate samples to investigate if the profile of CCR7 mRNA and protein expression correlated with the ability of MDDC to migrate to a CCL19 concentration gradient, measured 48 h post-infection (Fig. 5D, n = 5 donors). Indeed, secondary stimulation with LPS induced a strong and significant (p≤0.05) increase of migration of rgHMPV- and rgHRSV-stimulated MDDC as compared to virus-treated cells given a mock secondary treatment. Following secondary stimulation of virus-treated cells with the cocktail of pro-inflammatory cytokines, there was an increase in migration of mock-, rgHMPV- and rgHRSV-stimulated MDDC, although this did not reach statistical significance, and did not reach the level of increase induced by LPS. Taken together, these results suggest that the low concentration of TNF-α, IL-1α and IL-6 induced by rgHMPV and rgHRSV is partly responsible for the low CCR7 mediated migration.

### Chemokine receptor expression in GFP-positive versus GFP-negative virus-stimulated MDDC following secondary stimulation with a cocktail of pro-inflammatory cytokines or LPS

**Figure 6:**
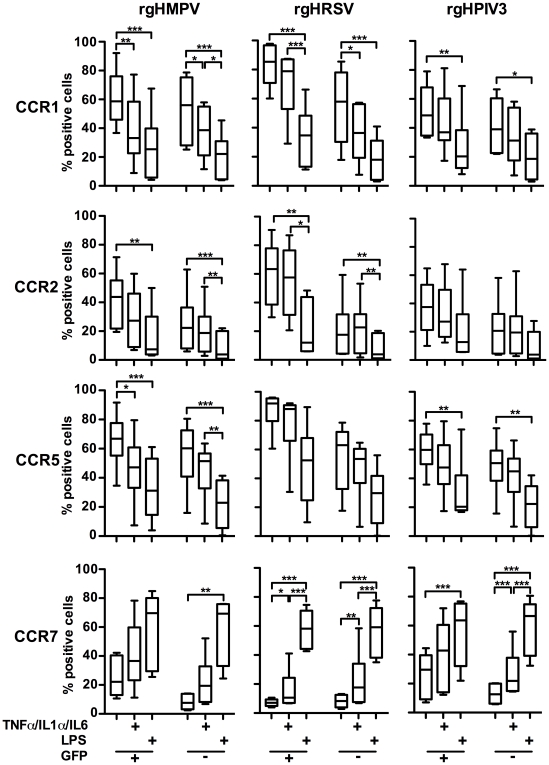
Surface expression of chemokine receptors on GFP-positive and GFP-negative MDDC after inoculation with rgHMPV, rgHRSV and rgHPIV3 and secondary stimulation with the pro-inflammatory cytokines TNF-α/IL-1α/IL-6 or with LPS. 4–6 h after infection of immature MDDC with rgHMPV or rgHRSV, cells were stimulated with a cocktail of the pro-inflammatory cytokines TNF-α/IL-1α/IL-6 or LPS. 48 h after the initial infection, GFP-positive and GFP-negative cells were analyzed by flow cytometry for cell surface expression of the indicated chemokine receptors. n = 6 donors. (+) GFP+ population, (−) GFP− population. The box plots show the median (horizontal line), flanked by the 2^nd^ and 3^rd^ quartile. The outers bars show the range of values. Statistical differences between relevant groups, i.e. with and without cytokine treatment, are indicated by asterisks (* p≤0.05, **p≤0.01, *** P≤0.001, see materials and methods).

We next investigated possible effects of robust viral infection (indicated by intracellular GFP expression) on chemokine receptor expression following treatment with the pro-inflammatory cytokine cocktail or LPS. This was done by infecting MDDC (n = 6 donors) with rgHMPV, rgHRSV, or rgHPIV3, subjecting them to a secondary stimulation with the pro-inflammatory cytokine cocktail or LPS at 4 h post-infection, and using flow cytometry to analyze the cell surface expression of CCR1, 2, 5, and 7 in the GFP-positive versus the GFP-negative populations at 48 h post-infection (Fig. 6).

Secondary stimulation of rgHMPV-, rgHRSV-, or rgHPIV3-stimulated MDDC with LPS decreased cell surface expression of CCR1, 2, and 5 on both GFP+ and GFP− cells. Secondary stimulation with the cocktail of pro-inflammatory cytokines also induced a decrease in surface expression of CCR1, 2, and 5. However, the magnitude of the effect usually was less than that observed with LPS.

Secondary stimulation of rgHMPV-, rHRSV-, or rgHPIV3-exposed MDDC with LPS induced an equally strong increase of CCR7 surface expression on GFP+ and GFP− cells, compared to cells that did not receive the secondary treatment (Fig. 6). Secondary stimulation of virus-infected cells with the pro-inflammatory cocktail also induced increases in CCR7 expression on both GFP− and GFP+ cells, although only in the case of rgHRSV GFP−+ and GFP− cells and rgHPIV3 GFP− cells was this difference statistically significant compared to cells receiving a mock secondary treatment. This provided further evidence that the poor expression of CCR7 in MDDC exposed to rgHRSV or rgHMPV could be overcome by secondary stimulation with LPS, and substantially overcome by secondary stimulation with the cocktail of pro-inflammatory cytokines. These increases were observed both in GFP+ and GFP− cells, indicating that robust viral infection did not irreversibly block CCR7 expression.

## Discussion

Compared to HPIV3 or IAV, stimulation of human MDDC with HRSV or HMPV *in vitro* resulted in inefficient maturational changes in chemokine receptor usage – namely down-regulation of CCR1, CCR2, and CCR5 and up-regulation of CCR7 – that are necessary for DC migration *in vivo* following antigen uptake. MDDC stimulated with HRSV or HMPV did not migrate efficiently towards a CCL19 gradient in an *in vitro* assay, compared to HPIV3 or IAV, confirming that the poor surface expression of CCR7 had functional consequences. The weak chemokine receptor modulation and migration by MDDC exposed to HMPV and HRSV, viruses that are thought to induce incomplete immunity, was particularly evident compared to MDDC exposed to IAV, a virus that induces effective immunity.

*In vivo*, maturing, antigen-bearing DC migrate from peripheral tissue to secondary lymphatic tissue and localize in defined lymphoid compartments, where they present antigens to CD4+ and CD8+ T lymphocytes, initiating and polarizing the T cell response [26], [50]. DC migration to and positioning within lymphatic tissue are critical towards mounting an effective adaptive immune response [50]. While there are multiple chemokine receptors that direct immature DCs towards peripheral sites, CCR7 is the only receptor that mediates migration toward and positioning within lymphatic compartments for interaction with T lymphocytes [30], [51], [52], [53]. Thus, differential effects of pathogens on CCR7 expression in particular could be functionally relevant for differences in the immune response to these pathogens. Accordingly, the reduced migration observed in our *in vitro* assay for HMPV- and HRSV-treated MDDC following stimulation with HRSV and HMPV suggests that, during an HMPV or HRSV infection *in vivo*, maturing DC migrate with reduced efficiency from the infected mucosa towards secondary lymphatic tissues. This might lead to reduced adaptive immune responses that could explain the greater ability of HMPV and HRSV to reinfect humans throughout life without need for significant antigenic change.

The present study was done with primary human cells from multiple donors. While the use of cells from an outbred population provides data with substantial individualistic differences and reduced statistical significance compared to convenient, uniform hosts like inbred mice, it is important to note that the natural host of the viruses in the present study is the human and not the mouse. Direct *in vivo* studies of virus-specific effects on DC migration during respiratory infections of humans are difficult, especially in children. Gill et al [54] noted that DC persisted in the lungs of children hospitalized for HRSV infection for as long as 8 weeks following the resolution of infection [55]. Resorting to data from mice, sustained increases in pulmonary DC have also been observed following HRSV infection [56]. Lucken et al [57] tracked the migration of mouse DC following HRSV infection and showed that the increase in DC numbers in the mouse mediastinal lymph node was slower compared to IAV or Sendai virus infection [58], [59], [60]. These observations would be consistent with inefficient migration from the lung to lymphoid tissue. Our *in vitro* studies now provide a mechanism for these previous *in vivo* observations. In addition, we provided data that MDDC maturation also was reduced with HMPV compared to HPIV3 and IAV.

We previously provided data indicating that the level of MDDC maturation in response to exposure to HMPV and HRSV is lower compared to HPIV3 [32] and IAV (not shown). *In vivo*, the combination of these two factors, namely reduced overall maturation and inefficient CCR7-CCL19 driven migration, might result in additive net effects that could affect both the magnitude and the quality of the adaptive immune response. Compared to infection with IAV, HRSV and HMPV infections may yield lower overall numbers of virus-stimulated mature DC in the afferent lymphatics. Reduced expression of co-stimulatory surface molecules and reduced cytokine expression could affect the quality of the response as well as its magnitude. In addition, the inefficient migration of maturing DCs may also play a role in viral pathogenesis: specifically, the sustained presence of mature DC in the mouse lung has been suggested to contribute to airway inflammation [56].

Another paramyxovirus, measles virus (MeV), was recently shown to inhibit CCR7-driven DC migration. Interference with DC maturation and function is considered to be central to MeV-induced immunosuppression. Compared to LPS, MeV infection failed to promote the switch from CCR5 to CCR7 expression, and MeV-matured DC exhibited chemotactic responses to CCL3 rather than to CCL19 [61]. Inhibition of CCR7-driven migration was also described for vaccinia virus and for herpes simplex virus type 1 [45], [62], [63]. However, the effects of reduced DC maturation and migration on long-term protection might be particularly significant for respiratory viruses such as HMPV and HRSV. Both of these viruses are restricted in tropism to the superficial cell layer of the respiratory tract, and protection against re-infection has reduced effectiveness (compared to viremic viruses, for example) due to the short-lived nature of local IgA antibodies, the inefficiency with which serum antibodies access the respiratory lumen, and the down-regulation of virus-specific CD8+ T cell functionality in the respiratory tract [64]. Thus, even modest decreases in the magnitude of the adaptive response could result in decreases in viral clearance and protection against re-infection.

We used recombinant GFP-expressing viruses to distinguish between effects in robustly infected (GFP-positive) and uninfected/abortively-infected (GFP-negative cells) MDDC. This revealed additional differences between the viruses. For MDDC infected with HMPV or HPIV3, the GFP-positive population expressed significantly more surface CCR7 than the GFP-negative population. In contrast, for MDDC infected with HRSV, the GFP-positive subpopulation resembled the GFP-negative population in having very low CCR7 surface expression. Thus, whereas robust infection with HMPV and HPIV3 stimulated expression of CCR7, robust infection with HRSV did not. Furthermore, GFP-positive cells infected with HRSV showed no down-regulation of CCR1, 2, and 5 surface expression. Thus, compared to HMPV or HPIV3, even the subpopulation of DC that is robustly infected with HRSV and contains abundant intracellular antigen would not be mobilized for migration. This would impede the delivery of HRSV antigen from the periphery to lymphoid tissue. Furthermore, DC that are robustly infected with a virus can readily process newly synthesized viral antigens for display on MHC class I molecules and presentation to CD8+ T cells. Reduced migration of DC that are robustly infected with HRSV to lymphoid tissue would reduce this activity. This would make activation of CD8+ T cells more dependent on cross-presentation by non-infected DC, and could reduce the efficiency of CD8+ T cell activation during HRSV infection, reducing viral clearance and the disease-sparing regulatory effects of HRSV-specific CD8+ T cells [65].

Secondary stimulation of HRSV- or HMPV-stimulated MDDC with LPS, a strong DC activator, resulted in up-regulation of CCR7 expression on both GFP-negative and GFP-positive cells and increased *in vitro* migration. In contrast, with vaccinia virus or human cytomegalovirus, a secondary stimulation of the infected DC with LPS failed to up-regulate the CCR7 chemokine receptor [45], [62]. LPS is a strong NFκ-B and AP-1 dependent DC activator [66], [67]. Secondary stimulation of HRSV- and HMPV-infected MDDC with the NFκ-B/AP-1-dependent pro-inflammatory cytokines TNF-α, IL-1α and IL-6, at concentrations comparable to those induced by LPS treatment, up-regulated CCR7 expression and was pro-migratory. This suggests that, in contrast to MeV, vaccinia virus, or herpes simplex virus, suboptimal stimulation, rather than inhibition, is responsible for the poor-migration phenotype of pneumovirus-exposed MDDC.

In summary, compared to HPIV3 and, in particular, IAV, the pneumoviruses HMPV and HRSV were inefficient in inducing the maturation-related changes in cell surface chemokine receptor expression in MDDC that are necessary *in vivo* to re-direct DC from the periphery to lymphoid tissue. Consistent with this, both HRSV and HMPV were poor inducers of MDDC maturation and migration *in vitro*. These effects could be contributing factors in the incomplete nature of protection induced by HRSV infection in humans.

## Supporting Information

Table S1 **Genes analyzed by TaqMan Gene-Expression Assay.** A low-density Taqman array representing 62 human genes was used for analysis of gene expression of MDDC stimulated with rHMPV, rHRSV, rHPIV3, or IAV; genes and Taqman assay numbers are listed in Table S1.
(DOC)

Figure S1 **Gene expression of MDDC stimulated with rHMPV, rHRSV, rHPIV3, or IAV.** Immature MDDC (n = 3 donors, numbered 1–3) were stimulated with SEB or infected with live or UV-inactivated rHMPV, rHRSV, rHPIV3, or influenza/A/Udorn (IAV). Twenty-four h post infection, total cellular RNA was prepared and reverse-transcribed using random primers, and the cDNA analyzed in triplicate by qPCR using a low-density Taqman array representing 62 human genes (see Table S1). The genes were grouped based on biological function: i) type I and III IFNs (n = 5), ii) transcription factors (n = 10); iii) pro-inflammatory cytokines (n = 6), (iv) Th1 cytokines (n = 5), (v) Th2 cytokines (n = 3), (vi) Th17/Tr-1 cytokines (n = 5), (vii) pattern recognition receptors and signaling intermediates (n = 9), (viii) maturation markers (n = 8), (ix) major histocompatibility (MHC) molecules (n = 6), and (x) chemokine receptors (n = 5). qPCR results were analyzed using the comparative threshold cycle (ΔΔC_T_) method, normalized to 18S rRNA. (A, B) The results are (A) expressed as log_2_ fold change over mock and presented as a heat map (scales shown to the left of each panel) with each group as a separate hierarchical cluster of log_2_ ratios (GENESIS program, release 1.7.2, http://genome.tugraz.at [65]), or (B) as fold-increase over mock for individual genes. Note that the rHMPV, rHRSV, and rHPIV3 viruses used in this experiment did not express GFP, whereas all subsequent experiments used GFP-expressing versions. Among the responsive donors, one notable difference among the viruses was the low type I/III IFN response to rHRSV: very low levels of IFN-α1 and IFN-β were induced, and there was no induction of IL-28A and IFN-α2. All four viruses induced the expression of transcription factors involved in orchestrating DC maturation, and innate immune response genes (IRF-7, IRF-1, and STAT-1). The transcription factors NFκ-B, STAT-3, and JAK-1 were also increased, albeit at a lower level, and with substantial donor-to-donor variability. Several pro-inflammatory chemokine genes, namely CCL8, CCL5, CXCL9, and CXCL10, were strongly up-regulated by rHMPV, rHPIV3, and IAV. For rHRSV, the non-responsive donor 2 and also donor 3 showed a limited response for these cytokines, which may be due to the low IFN induction by rHRSV. The Th1 associated genes IFN-α, CXCL9, CXCL10, IL-12A, and IL-18 were strongly up-regulated in response to all viruses, but the Th2 associated genes IL-4, CCL22, and CCL17 were not. All of the viruses induced IL-27A and, to some extent, TGF-β, IL-6, and IL1-β, suggesting that these MDDC might also be able to induce to some extent a regulatory T cell (Treg) response. However, IL-23 expression was variable between donors and appeared increased in donor 2, but decreased in donor 3, with little difference between rHPMV, rHRSV and rHPIV3, suggesting a variable Th-17 response. IL-23 was not induced in any donor in response to IAV. The viruses were similar with respect to induction of pattern recognition receptors and adapters. In particular, we detected a strong up-regulation of RIG-I and Mda5, as well as up-regulation of genes of the TLR pathways (TLR3 and its adaptor TRIF, TLR7 and TLR8 and their adaptor MyD88). However, rHPIV3 and IAV induced down-regulation (3- to 100-fold) of CD14, which is associated with TLR-4. All of the viruses also induced the up-regulation of typical cell surface maturation markers including CD38, CD40, CD80, CD86, MHC-class I, PDL-1 and PDL-2, although the response tended to be reduced with rHRSV.
(TIF)
