# Supplementary material for: Low CCR7-Mediated Migration of Human Monocyte Derived Dendritic Cells in Response to Human Respiratory Syncytial Virus and Human Metapneumovirus
Source: PLoS Pathog. 2011 Jun 23;7(6):e1002105. doi: 10.1371/journal.ppat.1002105 (PMC3121884; doi:10.1371/journal.ppat.1002105)
Supplement: Table S1 — Genes analyzed by TaqMan Gene-Expression Assay. A low-density Taqman array representing 62 human genes was used for analysis of gene expression of MDDC stimulated with rHMPV, rHRSV, rHPIV3, or IAV; genes and Taqman assay numbers are listed in Table S1. (DOC) [file ppat.1002105.s001.doc]

**Table S1: Genes Analyzed by TaqMan Gene-Expression Assay**

|  | **Gene** | **ABI assay number^a^** |
| --- | --- | --- |
| **Type I, III IFN genes** | IFN-β1 | Hs01077958_s1 |
|  | IFN-α1 | Hs00256882_s1 |
|  | IFN-α2 | Hs00265051_s1 |
|  | IL-28A | Hs00820125_g1 |
|  | IL-29 | Hs00601677_g1 |
| **Transcription factors** | IRF7 | Hs00185375_m1 |
|  | SOCS1 | Hs00864158_g1 |
|  | IRF1 | Hs00971960_m1 |
|  | STAT1 | Hs00234829_m1 |
|  | NFKB2 | Hs00174517_m1 |
|  | NFKB1 | Hs00231653_m1 |
|  | STAT3 | Hs00374280_m1 |
|  | JAK1 | Hs00233820_m1 |
|  | IRF3 | Hs01547282_m1 |
|  | SARM1 | Hs00248344_m1 |
| **Cytokine genes** | CCL8 | Hs99999026_m1 |
|  | CCL5 | Hs00174575_m1 |
|  | TNF-α | Hs00174128_m1 |
|  | IL-10 | Hs00174086_m1 |
|  | IL-21 | Hs00222327_m1 |
|  | IL-12B | Hs01011518_m1 |
| **Th1 cytokine genes** | CXCL9 | Hs00171065_m1 |
|  | CXCL10 | Hs00171042_m1 |
|  | IFN-γ | Hs00989291_m1 |
|  | IL-12A | Hs00168405_m1 |
|  | IL-18 | Hs99999040_m1 |
| **Th2 cytokine genes** | IL-4 | Hs00174122_m1 |
|  | CCL22 | Hs99999075_m1 |
|  | CCL17 | Hs00171074_m1 |
| **Th17 / Treg cytokine genes** | IL-27A | Hs00377366_m1 |
|  | IL-6 | Hs00174131_m1 |
|  | IL-1β | Hs00174097_m1 |
|  | TGF-β1 | Hs00998130_m1 |
|  | IL-23A | Hs00372324_m1 |
| **Pattern recognition receptor genes** | RIG-I | Hs00204833_m1 |
|  | Mda5 | Hs01070332_m1 |
|  | TRIFF | Hs00706140_s1 |
|  | MyD88 | Hs00182082_m1 |
|  | TLR7 | Hs00152971_m1 |
|  | TLR3 | Hs00152933_m1 |
|  | TLR8 | Hs00152972_m1 |
|  | TLR4 | Hs00152939_m1 |
|  | CD14 | Hs00169122_g1 |
| **Maturation marker genes** | CD38 | Hs00233552_m1 |
|  | CD80 | Hs00175478_m1 |
|  | CD40 | Hs00374176_m1 |
|  | PD-L1 | Hs00204257_m1 |
|  | PD-L2 | Hs00228839_m1 |
|  | CD54 | Hs00164932_m1 |
|  | CD86 | Hs99999104_m1 |
|  | DC-SIGN | Hs01588349_m1 |
| **MHC genes** | HLA-A | Hs01058806_g1 |
|  | HLA-B | Hs00741005_g1 |
|  | HLA-C | Hs03044135_m1 |
|  | HLA-DPA1 | Hs00410276_m1 |
|  | HLA-DQB1 | Hs03054971_m1 |
|  | HLA-DRB1 | Hs99999917_m1 |
| **Chemokine receptor genes** | CCR7 | Hs00171054_m1 |
|  | CCR3 | Hs00266213_s1 |
|  | CCR1 | Hs00174298_m1 |
|  | CCR5 | Hs00152917_m1 |
|  | CCR8 | Hs00174764_m1 |
| **Endogenous control** | 18S RNA | Hs99999901_s1 |

^a^Applied Biosystems gene expression assay number
