# Supplementary figures and images for: Low CCR7-Mediated Migration of Human Monocyte Derived Dendritic Cells in Response to Human Respiratory Syncytial Virus and Human Metapneumovirus
Source: PLoS Pathog. 2011 Jun 23;7(6):e1002105. doi: 10.1371/journal.ppat.1002105 (PMC3121884; doi:10.1371/journal.ppat.1002105)

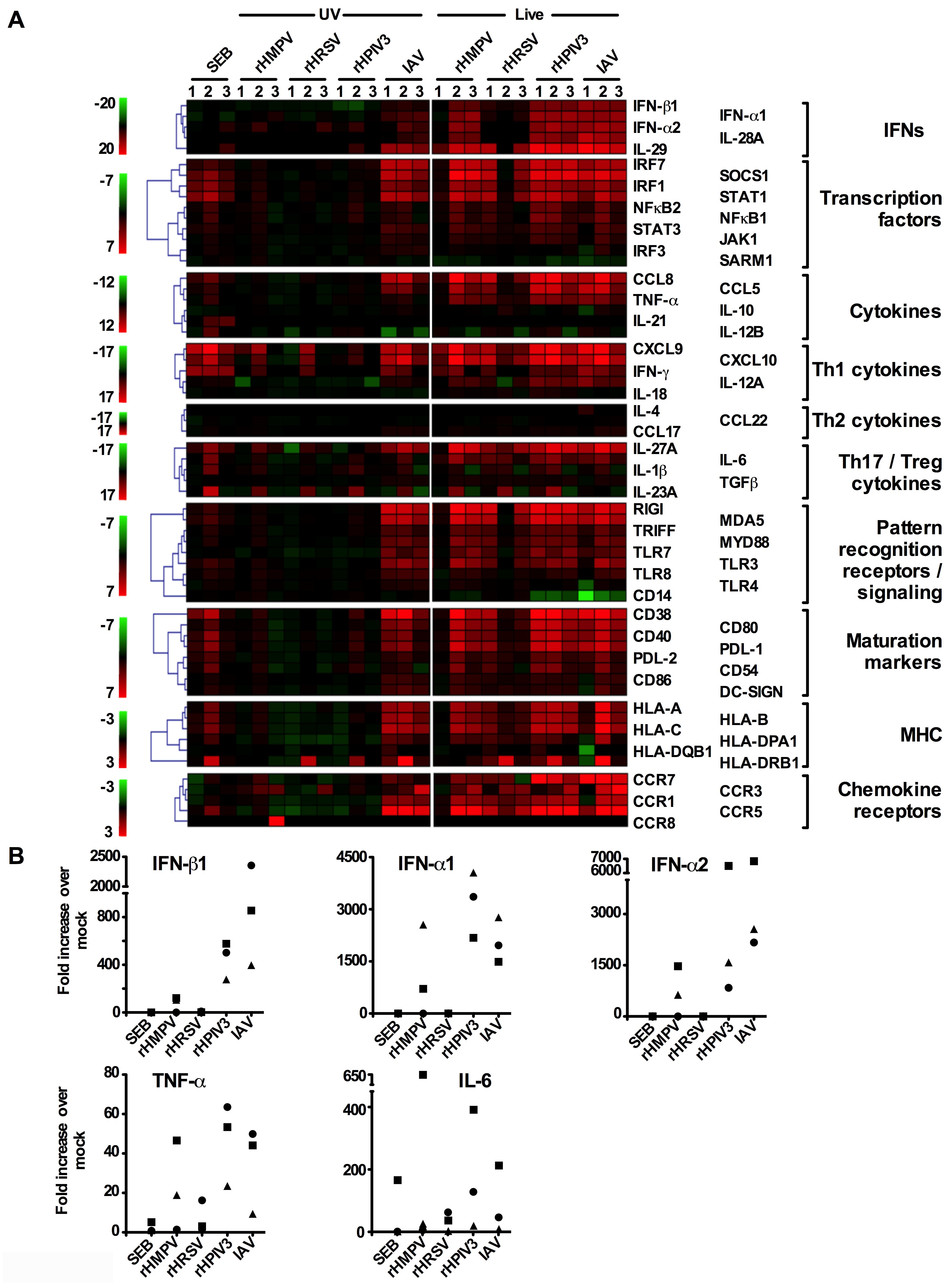

Supplement: Figure S1 — Gene expression of MDDC stimulated with rHMPV, rHRSV, rHPIV3, or IAV. Immature MDDC (n = 3 donors, numbered 1–3) were stimulated with SEB or infected with live or UV-inactivated rHMPV, rHRSV, rHPIV3, or influenza/A/Udorn (IAV). Twenty-four h post infection, total cellular RNA was prepared and reverse-transcribed using random primers, and the cDNA analyzed in triplicate by qPCR using a low-density Taqman array representing 62 human genes (see Table S1). The genes were grouped based on biological function: i) type I and III IFNs (n = 5), ii) transcription factors (n = 10); iii) pro-inflammatory cytokines (n = 6), (iv) Th1 cytokines (n = 5), (v) Th2 cytokines (n = 3), (vi) Th17/Tr-1 cytokines (n = 5), (vii) pattern recognition receptors and signaling intermediates (n = 9), (viii) maturation markers (n = 8), (ix) major histocompatibility (MHC) molecules (n = 6), and (x) chemokine receptors (n = 5). qPCR results were analyzed using the comparative threshold cycle (ΔΔCT) method, normalized to 18S rRNA. (A, B) The results are (A) expressed as log2 fold change over mock and presented as a heat map (scales shown to the left of each panel) with each group as a separate hierarchical cluster of log2 ratios (GENESIS program, release 1.7.2, http://genome.tugraz.at [65]), or (B) as fold-increase over mock for individual genes. Note that the rHMPV, rHRSV, and rHPIV3 viruses used in this experiment did not express GFP, whereas all subsequent experiments used GFP-expressing versions. Among the responsive donors, one notable difference among the viruses was the low type I/III IFN response to rHRSV: very low levels of IFN-α1 and IFN-β were induced, and there was no induction of IL-28A and IFN-α2. All four viruses induced the expression of transcription factors involved in orchestrating DC maturation, and innate immune response genes (IRF-7, IRF-1, and STAT-1). The transcription factors NFκ-B, STAT-3, and JAK-1 were also increased, albeit at a lower level, and with substantial donor-to- [file ppat.1002105.s002.tif]
